# Supplementary material for: Towards ubiquitous radio access using nanodiamond based quantum receivers
Source: Commun Eng. 2025 Mar 31;4:60. doi: 10.1038/s44172-025-00396-4 (PMC11958799; doi:10.1038/s44172-025-00396-4)
Supplement: Supplementary file 2 — Supplementary Information [file 44172_2025_396_MOESM2_ESM.pdf]

Supplementary Information

Towards ubiquitous radio access using nanodiamond based quantum receivers

Qunsong Zeng[1,†], Jiahua Zhang[1,†], Madhav Gupta[1], Zhiqin Chu[1,2,*], Kaibin Huang[1,*]

[†] Equal contribution

[*] Corresponding authors (Email: zqchu@eee.hku.hk; huangkb@eee.hku.hk)

[1] Department of Electrical and Electronic Engineering, The University of Hong Kong, Hong Kong, China

[2] School of Biomedical Sciences, The University of Hong Kong, Hong Kong, China

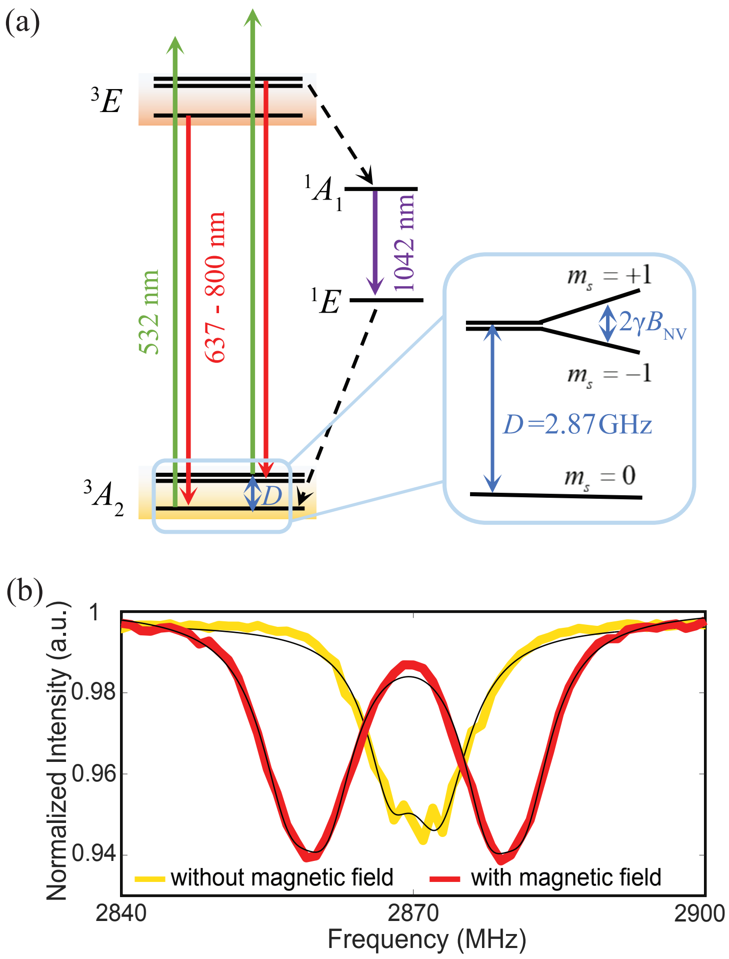

Supplementary Fig. 1. Nitrogen-vacancy (NV) center energy levels and the optically detected magnetic resonance (ODMR) spectrum. **(a)** The NV ground-state spin sub-levels are separated by D = 2.87 GHz. Upon optical excitation at 532 nm by a green laser, the population of the ground state can be read out by monitoring the intensity of the emitted fluorescence light. With no static magnetic field, NV center ground states are degenerate. Applying an external static magnetic field causes a Zeeman shift, removing degeneracy and determining resonance frequencies using the formula $\nu_{\pm} = |D \pm \gamma B_{NV}|$. **(b)** ODMR spectrum of NV centers in diamond. The yellow and red curves are experimental results without and with external magnetic field, respectively. The thin black curves come from the smooth Lorentz curve fit.

Supplementary Note 1. Principle of Diamond Receiver for Supporting Multiple Access

In the past decade, a single diamond with nitrogen-vacancy (NV) centers has been studied as a microwave detector. However, in real-world communication systems, it is required to function as a receiver that can support multiple access. As an example, in a single-band and/or multi-band communication system, the NV-diamond receivers would be positioned at the base station to simultaneously accommodate multiple users. To effectively demultiplex signals from multiple users, the receiver is expected to have the capability to distinguish between the combinations of different microwave signals transmitted by these users.

To demonstrate the diamond's ability to function as a multi-user receiver, we utilized a single diamond with two users as an example. To showcase the feasibility of the receiver, we employed continuous wave (CW) optically detected magnetic resonance (ODMR), which is a simple but robust method to detect the interactions between diamond NV-centers and microwaves. The fluorescence photoluminescence (PL) of NV centers, with a Lorentzian line shape, is given by

$$PL = \Lambda_0 \left[1 - C \frac{1}{1 + \left(\frac{f - f_0}{\Gamma} \right)^2} \right],$$

where Λ_0 is the cumulative off-resonance PL, C is the ODMR contrast, f_0 is the resonance frequency and Γ is the half-width at half maximum (HWHM). The contrast is dependent on the Rabi frequency and laser power [1]:

$$C \propto \frac{\Omega_R^2}{\Omega_R^2 + \Gamma_p^\infty \Gamma_c^\infty \left(\frac{P_{opt}}{P_{opt} + P_{sat}} \right)^2},$$

where Γ_p^∞ is the polarization rate at saturation, Γ_c^∞ is the rate of optical cycles at saturation, P_{opt} is optical pumping power, P_{sat} is the saturation power, and Ω_R is Rabi frequency. Especially, Rabi frequency has a relationship with the incident microwave: $\Omega_R \propto \vec{B}_{mwp}$, where $\vec{B}_{mwp}$ is the component of the vector microwave magnetic field $\vec{B}_{mw}$ that is perpendicular to the axis of electron spin [2]. The relationship between $\vec{B}_{mwp}$ and $\vec{B}_{mw}$ is given by

$$\vec{B}_{mwp} = \vec{B}_{mw} \sin\theta,$$

where θ is the angle between the vector microwave magnetic field $\vec{B}_{mw}$ and the axis of electron spin. In a multiple access communication system, two users transmit information using frequency digital modulation and map bits 0 and 1 to two microwave frequencies: one on resonance f_0 and the other off resonance f_1. The two users are situated at different distances (r_0, r_1), and transmit microwave frequencies with the same amplitude $\vec{B}_{mw}$ to the NV centers within the bulk diamond,

resulting two angles (θ_0, θ_1).

There are four reference bit pairs predetermined to realize all possible combinations of bits from the two users. The results are shown in the Supplementary Table 1.

Supplementary Table 1. Reference bit pairs for multiple access communication system

The reference bit pairs	Transmitted frequency pairs	Reached microwave amplitude	PL Intensity*
(0,0)	$(f_0(r_0), f_0(r_1))$	$\vec{B}_{mw0}(r_0)\sin(\theta_0) + \vec{B}_{mw0}(r_1)\sin(\theta_1)$	$\Lambda_0\left(1 - C_{(f_0(r_0),f_0(r_1))}\right)$
(0,1)	$(f_0(r_0), f_1(r_1))$	$\vec{B}_{mw0}(r_0)\sin(\theta_0) + \vec{B}_{mw1}(r_1)\sin(\theta_1)$	$\Lambda_0\left(1 - C_{f_0(r_0)} - C_{f_1(r_1)}\frac{1}{1+\left(\frac{f_1-f_0}{\Gamma}\right)^2}\right)$
(1,0)	$(f_1(r_0), f_0(r_1))$	$\vec{B}_{mw1}(r_0)\sin(\theta_0) + \vec{B}_{mw0}(r_1)\sin(\theta_1)$	$\Lambda_0\left(1 - C_{f_0(r_1)} - C_{f_1(r_0)}\frac{1}{1+\left(\frac{f_1-f_0}{\Gamma}\right)^2}\right)$
(1,1)	$(f_1(r_0), f_1(r_1))$	$\vec{B}_{mw1}(r_0)\sin(\theta_0) + \vec{B}_{mw1}(r_1)\sin(\theta_1)$	$\Lambda_0\begin{pmatrix}1 - C_{(f_1(r_0),f_1(r_1))} \\ \times\frac{1}{1+\left(\frac{f_1-f_0}{\Gamma}\right)^2}\end{pmatrix}$

* $\vec{B}_{mw0}(r_0)\sin(\theta_0) + \vec{B}_{mw0}(r_1)\sin(\theta_1)$ is equal to one microwave magnetic field effect due to the same frequency, then the equal $\vec{B}_{equal}$ leads to the one contrast $C_{(f_0(r_0),f_0(r_1))}$. It is same for the case using off resonant microwave frequencies.

The four reference bit pairs mentioned above lead to four distinct fluorescence intensities at most angles, which means one diamond can serve as a receiver for two users. In the experiment, we swept the frequency range from 2846 to 2900 MHz, with each microwave amplitude retained around 0 dBm before being sent to the amplifier. The results are shown in Supplementary Fig. 2, and the PL intensity is calculated as the mean of the 100 × 100 pixels Region of Interest (RoI) in the widefield image, which has a total of 512 × 512 pixels. In the experimental setup, the vector microwave amplitudes were at the similar direction and the distance of user 1 was smaller than that of user 2. As a result, the arrived amplitude of microwave combinations in the NV-diamond has the relation: (0,0) > (0,1) > (1,0) > (1,1), so that the PL intensity from the NV centers has the order of (0,0) < (0,1) < (1,0) < (1,1). Experimentally, we map microwave frequencies: f_0 = 2860 MHz, f_1 = 2846 MHz, and the PL intensities are shown in the Supplementary Table 2. The experimental results in

Supplementary Table 2 verified the theory and proved the ability for NV-diamond as a receiver for supporting two users.

Supplementary Table 2. Reference bit pairs in the experiments

The reference bit pairs	(0,0)	(0,1)	(1,0)	(1,1)
PL Intensity (a.u.)	14540.1	14842.1	16006.2	16317.1

To illustrate the information transmission in such communication system, we conducted further experiments using two bulk diamonds. The usage of two bulk diamonds achieved a higher signal-to-noise ratio (SNR). As shown in Supplementary Fig. 3a, each user transmits a black-and-white image message, where each pixel is represented by 1 bit. The frame structure includes pilots (i.e., reference bits) and data payload (i.e., information bits). In this experiment, we map bits 0 and 1 to frequencies 2870 MHz and 2900 MHz, respectively (see Supplementary Fig. 3b). The two users (transmitters) simultaneously radiate microwaves, each of whose frequencies represents the individual bit stream. The resulting fluorescence intensities at two diamonds, induced by the pairs of reference bits, are shown in Supplementary Fig. 3c. These represent the effects of bit-pairs (0,0), (0,1), (1,0), and (1,1). Due to the inverse of two vector microwave magnetic fields, the intensity of bit pairs has the order of (1,0) < (0,1) < (0,0) < (1,1). Each NV-diamond reacts differently to different bit-pairs, with distinguishable intensity levels. It is also observed that one diamond is more discernible than the other, suggesting that more NV centers offer higher reliability for signals detection and demultiplexing. The transmission of data bits causes the fluorescence intensities variations as shown in Supplementary Fig. 3d, and the demultiplex of them induces the recovered images at the receiver as shown in Supplementary Fig. 3e.

As discussed above, one or two diamonds have limited capacity to support more users. To support more users with high reliability, abundant NV-diamonds with diversities are needed. Fluorescence nano-diamonds (FNDs), which are smaller in size than bulk diamonds, are the optimal choice as receivers. When randomly distributed on a cover glass, they can provide sufficient freedom of diverse spin orientations (parameter θ) and the number of NV centers in FNDs (parameter SNR). In addition, microwaves from different users encounter unique wireless channels (parameter r), further contributing to the system's ability to differentiate signals. Consequently, the responses of FNDs differ for all combinations of incident signals, allowing the system to distinguish and process signals from multiple users simultaneously.

In addition to simultaneously transmitting the 8-bit/pixel images to FNDs receivers as presented in the main text, we have also conducted transmission with black-and-white images, as shown in Supplementary Fig. 4. This additional experiment makes the bit errors in the recovered images more explicit to observe.

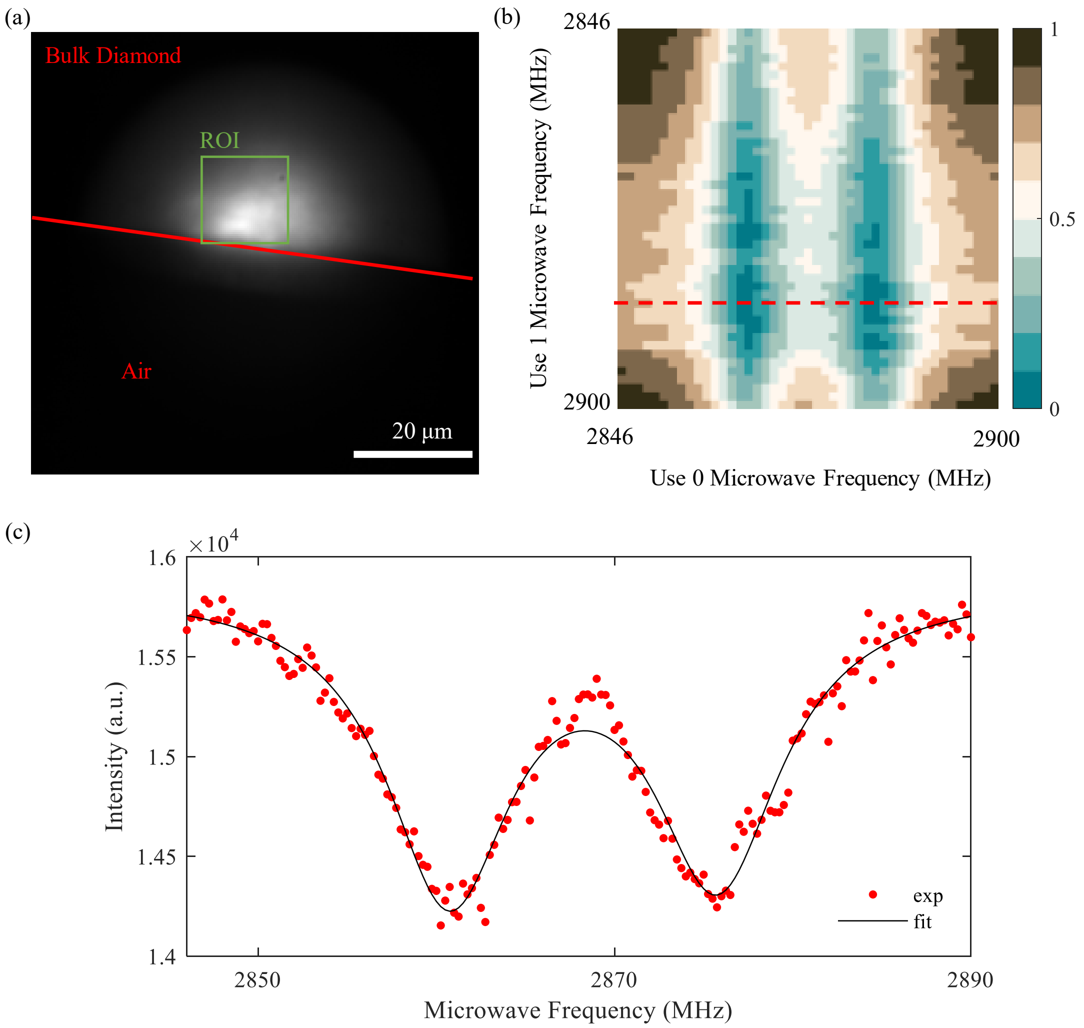

Supplementary Fig. 2. The ODMR spectrum of one bulk diamond under the sweep of two microwave frequencies. **(a)** The image of sample wide field. The ROI for calculating the mean PL intensity is labelled in green rectangle. **(b)** The two-dimensional map of the normalized sweep PL intensity. **(c)** The one-dimensional ODMR data comes from the red line in (b). The ODMR contrast is approximately 9%.

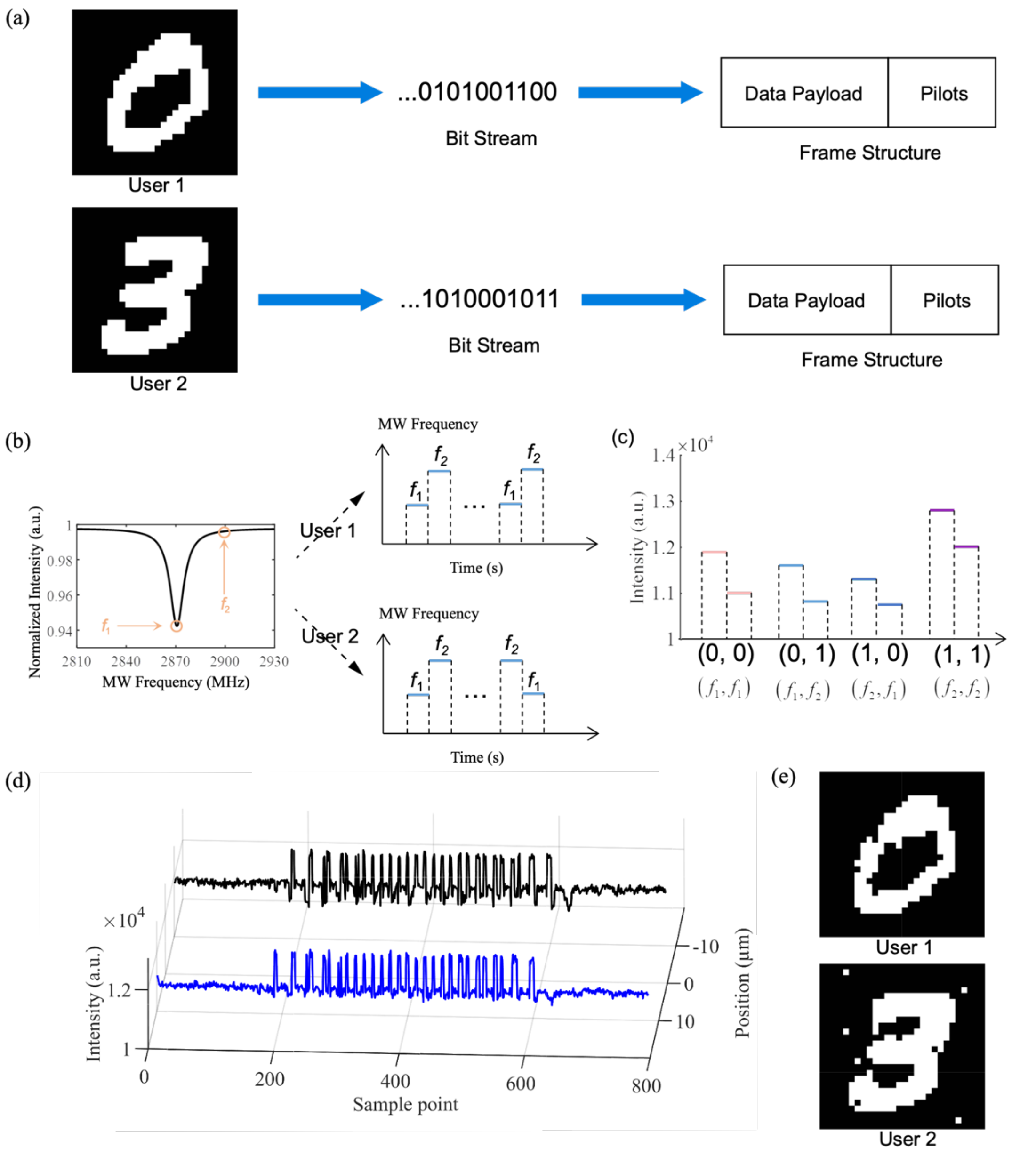

Supplementary Fig. 3. Experimental results using two bulk NV-diamonds. **(a)** The two black-and-white image messages are converted into two-bit streams, with each bit representing one pixel, and then combined with reference bits to create the frames. **(b)** Bit streams are modulated to frequencies 2870 MHz and 2900 MHz, corresponding to bits 0 and 1, respectively. **(c)** The resulting fluorescence intensities in response to all combinations of bit-pairs; for example, the two bars above (0,0) represent the intensities of the two diamonds in response to frequencies (f_1, f_1), respectively. **(d)** The resulting fluorescence intensities in response to bit-pair streams of images, with blue and black sequences representing the responses of the two bulk NV-diamonds. **(e)** The recovered image messages derived from the received intensities by comparing them with reference intensity levels.

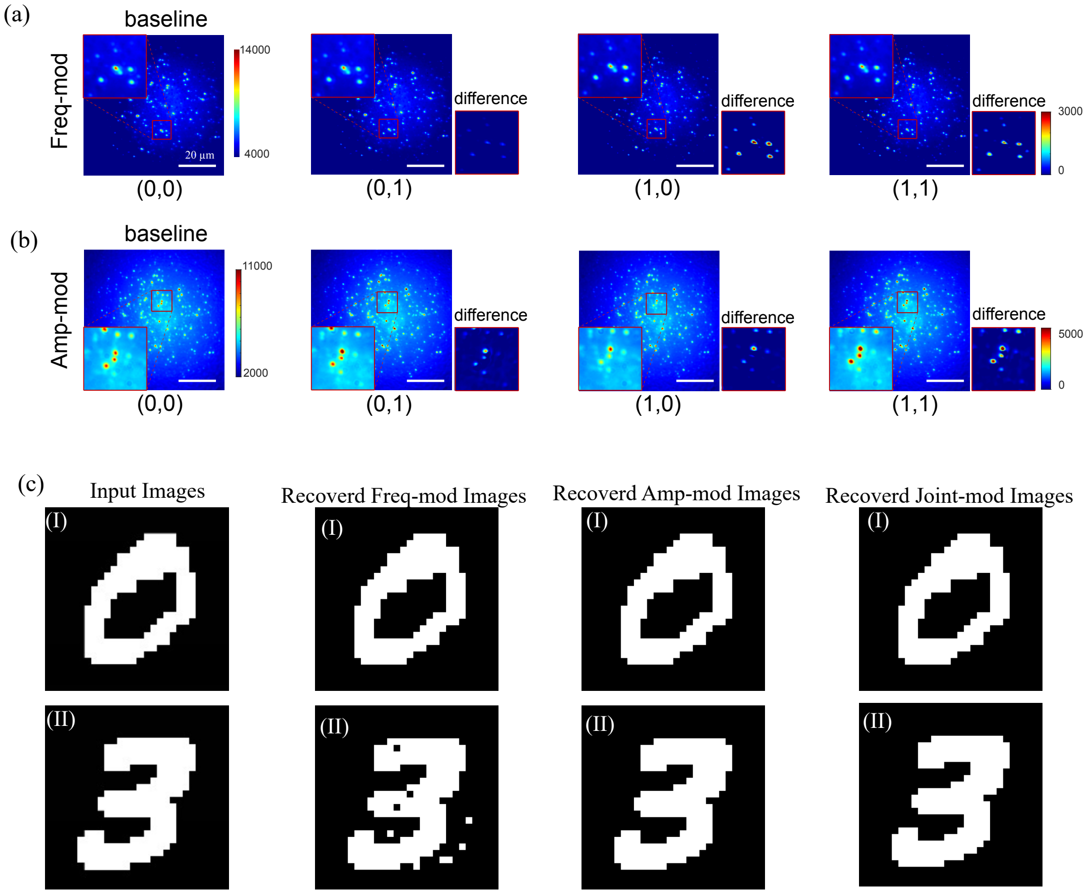

Supplementary Fig. 4. Additional experimental demonstrations of three multiple access systems using fluorescence nano-diamonds (FNDs)-based receiver. **(a)** Reference images obtained in the frequency modulation experiment. The baseline corresponds to the fluorescence intensity matrix when both users simultaneously transmit bit “0”. The other three images represent the remaining three-bit combinations, along with their differences from the baseline. **(b)** Reference images acquired in the amplitude modulation experiment, with the same interpretation as previously described. **(c)** Experimental results: input image messages; recovered image messages from the three experiments of frequency modulation, amplitude modulation, and joint frequency-and-amplitude modulation.

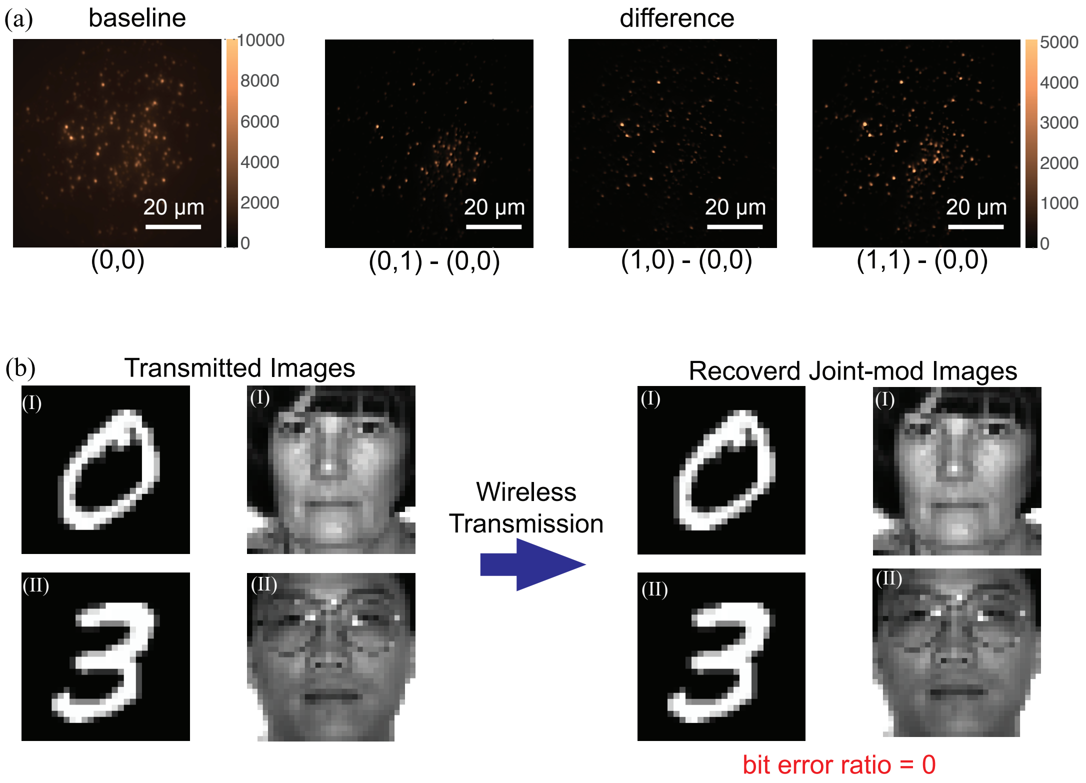

Supplementary Fig. 5. Experimental results of joint frequency-and-amplitude modulation. **(a)** Reference images obtained in the joint frequency-and-amplitude modulation experiment. The baseline corresponds to the fluorescence intensity matrix when both users simultaneously transmit bit "0". The other three images represent the remaining three-bit combinations, with their differences from the baseline. **(b)** (Left) Images transmitted by two users, images received during the joint modulation experiment, and (Right) images received during the experiment.

Supplementary Note 2. Working Principle of Reference-Free Design

In the main text, Fig. 4c illustrates that applying an external static magnetic field to FNDs results in distinct Lorentzian peaks in the ODMR spectrum. This distinction arises from the presence of various axes in the randomly distributed FNDs and the existence of a magnetic field gradient. As a result, different FNDs can respond to unique frequencies of incident microwaves.

To support multiple users transmitting data using distinct frequencies, K distinguishable FNDs are required to respond to K distinct microwave frequencies. This is feasible, given the typically large number of FNDs and the applied strong magnetic field, providing high capacity to support a significant number of users in a multiple access system (Supplementary Fig. 6).

Before transmitting information, pre-measured ODMR properties of all FNDs are needed to determine the possible frequency channels for use. Then, the concurrently transmitted signals can be detected and demultiplexed by monitoring the fluorescence intensity changes in these FNDs. This approach eliminates the need for transmitting reference bits to identify the fluorescence patterns of all possible combinations and consequently reduces communication overhead.

In the main text, we have demonstrated the feasibility of a reference-free design to serve two users simultaneously.

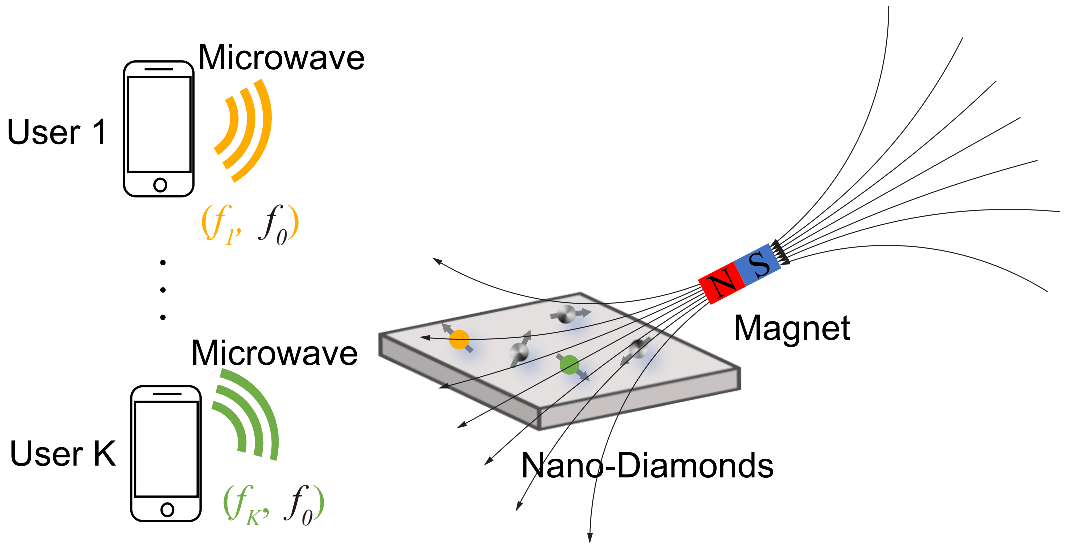

Supplementary Fig. 6. Illustration of the FNDs-based receiver for reference-free multiple signals detection and demultiplexing. The external static magnetic field is applied to enable different FNDs response to different microwave frequencies due to the existence of different axes of randomly distributed FNDs and the external magnetic field gradient.

Supplementary Note 3. Magnetic Field Gradient Measurement

We applied a bulk diamond to measure the magnetic field gradient for the magnetic field condition in Fig. 4. The wide field fluorescence image is shown in Supplementary Fig. 7a. We placed a magnetic field near the bulk diamond and found that it happened to be aligned with one NV axis in the bulk diamond, resulting in only four frequency peaks in the ODMR spectrum as shown in Supplementary Fig. 7b. We fitted the ODMR spectrum for each pixel and extracted the frequency peaks around 2815MHz to create the image of frequency peaks for all positions as shown in Supplementary Fig. 7c. The field of view for the images in Supplementary Fig. 7a and 7c is approximately 50 μm × 50 μm, and the frequency peak gradient is around 3.2 MHz/μm. We calculated the magnetic field gradient as approximately 0.023 G/um after dividing the gyromagnetic ratio of the electron spin γ = 2.8025 MHz/Gauss.

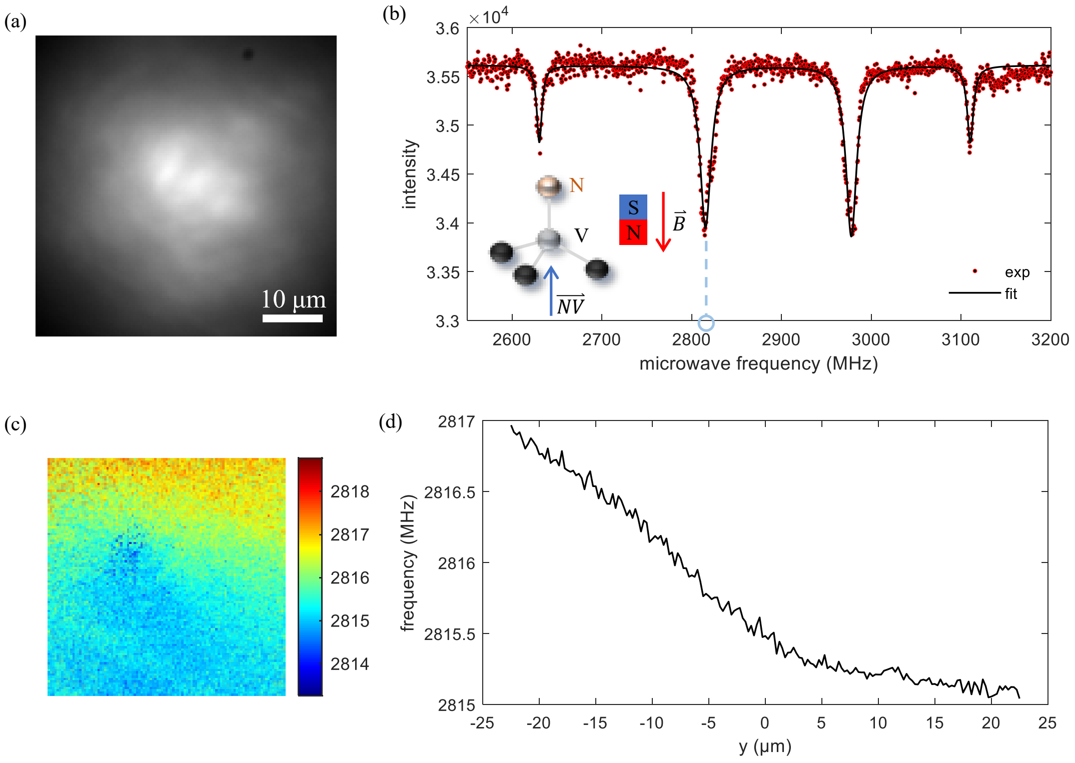

Supplementary Fig. 7. Magnetic field gradient measurement. **(a)** Wide field fluorescence image for a bulk NV-diamond. **(b)** The ODMR curve under the condition that the magnetic field align with one NV center axis in the diamond. **(c)** The image of frequency peak labeled in the bule circle in (b), calculated from the widefield image. **(d)** The curve reveals the relation between frequency peak and the from distance along y-axis.

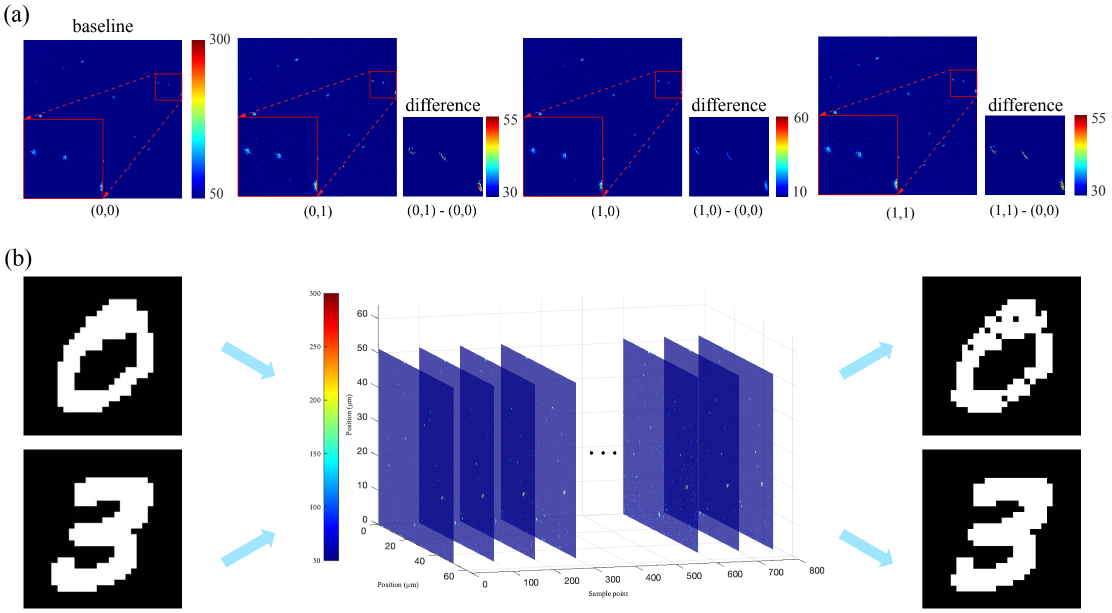

Supplementary Fig. 8. Experimental results using the implemented compact FNDs-based receiver device. **(a)** Received fluorescence images from reference bit-pairs in the experiment. The baseline corresponds to the fluorescence intensity matrix when both users simultaneously transmit bit “0”. The other three images represent the remaining three bit combinations, along with their differences from the baseline. **(b)** The left and right image pairs, transmitted and received image messages respectively, have been present in the Fig. 5c in main text. (Middle) Received fluorescence images from data bit-pairs.

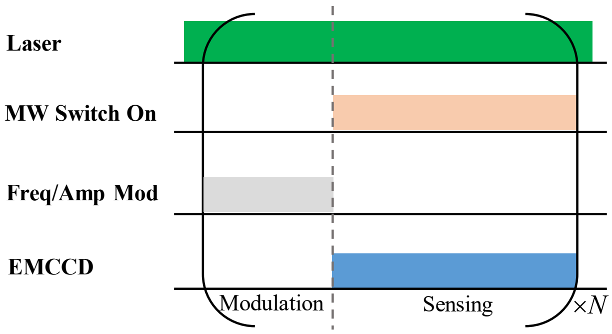

Supplementary Fig. 9. Pulse sequence of microwave detection using CW ODMR. The frequency or amplitude modulated signals are triggered from microwave source. The trigger time is set as 5 ms in the demonstration. The trigger time for microwave switch on and Electron-Multiplying CCD (EMCCD) is the same, around 30 ms.

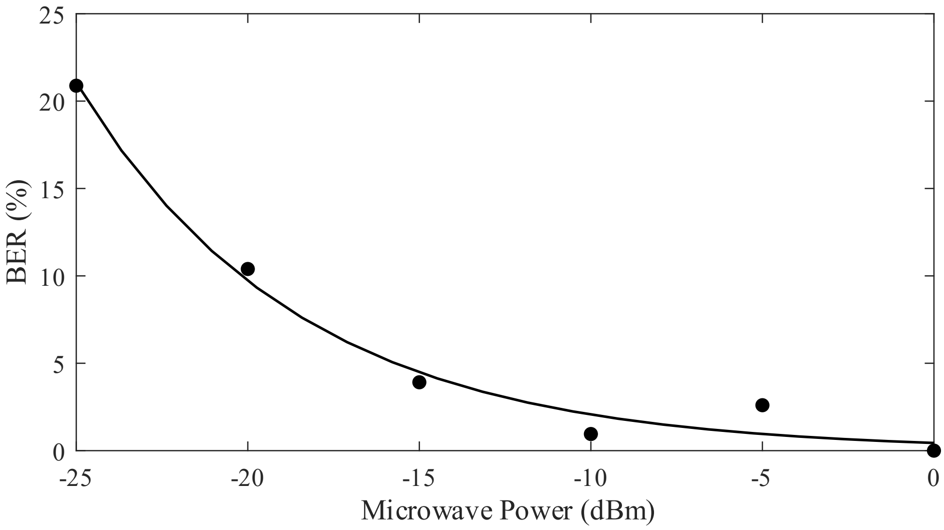

Supplementary Fig. 10. The relationship between signal strength (microwave power) and BER.

When the microwave power is set to -25 dBm, it is amplified to 5 dBm at the input of the microwave structure. The gold line within the microwave structure will emit -31 dBm of power, with the remaining power being transmitted to the end of the structure and some energy being lost as heat. This results in an emitted power of -31 dBm. After accounting for transmission losses, the nanodiamonds receive a power of -47.2 dBm. When the microwave power is set to 0 dBm, it is amplified to 30 dBm at the input of the microwave structure. The gold line within the microwave structure will emit -6 dBm of power, with the remaining power being transmitted to the end of the structure and some energy being lost as heat. This results in an emitted power of -6 dBm. After transmission, the nanodiamonds receive a power of -22.2 dBm.

For improvement, adding a low-noise amplifier (LNA) at the receiver's front end can significantly enhance sensitivity by reducing the system's effective noise figure (NF). Advanced signal processing techniques like matched filtering and adaptive filtering can further improve SNR by suppressing noise and enhancing signals.

Supplementary Note 4. Sensitivity Analysis

For an FND-receiver, nanodiamonds are utilized to detect microwave magnetic fields. In our setup, the sensitivity is determined as follows. We conducted ten experiments to calculate the sensitivity of FNDs when ODMR is applied for magnetic field detection. The frequency difference, denoted as Γ, between one peak and the center frequency was measured, as illustrated in Supplementary Fig. 11. The measurement gives the result that $\Gamma = 5.15 \pm 0.127$ MHz. Given the gyromagnetic ratio (γ) being 28 GHz/T, the measurement limit of the magnetic field intensity (ΔB) can be calculated as $\Delta B = \Delta\Gamma / \gamma = (0.127\ \mathrm{MHz}) / (28\ \mathrm{GHz/T}) = 4.536\ \mu\mathrm{T}$. The total ODMR measurement time was 38.099 s for whole 81 frequency points scan. Consequently, the sensitivity is determined using the equation $\eta_B = \Delta B/\sqrt{t} = (4.536\ \mu\mathrm{T})/\sqrt{(38.099\ \mathrm{s})} = 0.735\ \mu\mathrm{T} \cdot \mathrm{Hz}^{-1/2}$.

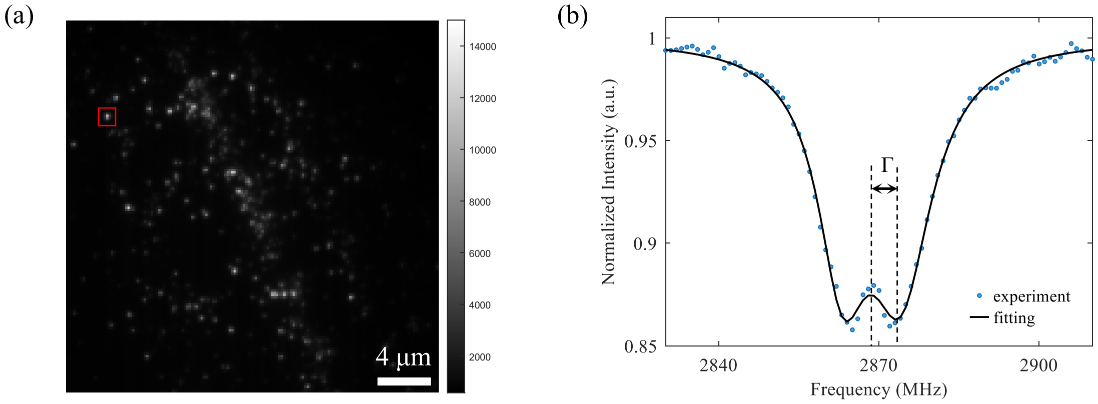

Supplementary Fig. 11. Measurements of FND sensitivity. **(a)** Microscopic image of FNDs, with the spots highlighted by the red rectangle selected for sensitivity analysis. **(b)** Key parameters utilized in the sensitivity calculation process.

Furthermore, this sensitivity has a theoretical limit value [1]:

$$\eta_B \approx 0.77 \frac{h}{g\mu_B} \frac{2\Delta\nu}{C\sqrt{R}}$$

where μ_B is the Bohr magneton, h is Planck constant, $g \approx 2$ for NV centers, C is the ODMR contrast, $\Delta\nu$ is the half-width at half maximum (HWHM), and R is photon counts. The contrast and HWHM are related to both the laser power and microwave power. We conducted experiments under varying laser and microwave power levels and plotted a map to analyze how these parameters influence the sensitivity. Because the camera intensity is not directly measured in photon counts, we cannot directly use the aforementioned formula to calculate the exact theoretical sensitivity. However, camera intensity is proportional to photon counts and thus there is a relationship for conversion between the two (though this relationship is difficult to determine precisely). We use the sum of the region of interest (ROI) intensity, I, in images as a substitute for R. We then plotted the map between $\Delta\nu/(C\sqrt{I})$ and the laser and microwave power, as shown in Supplementary Fig. 12.

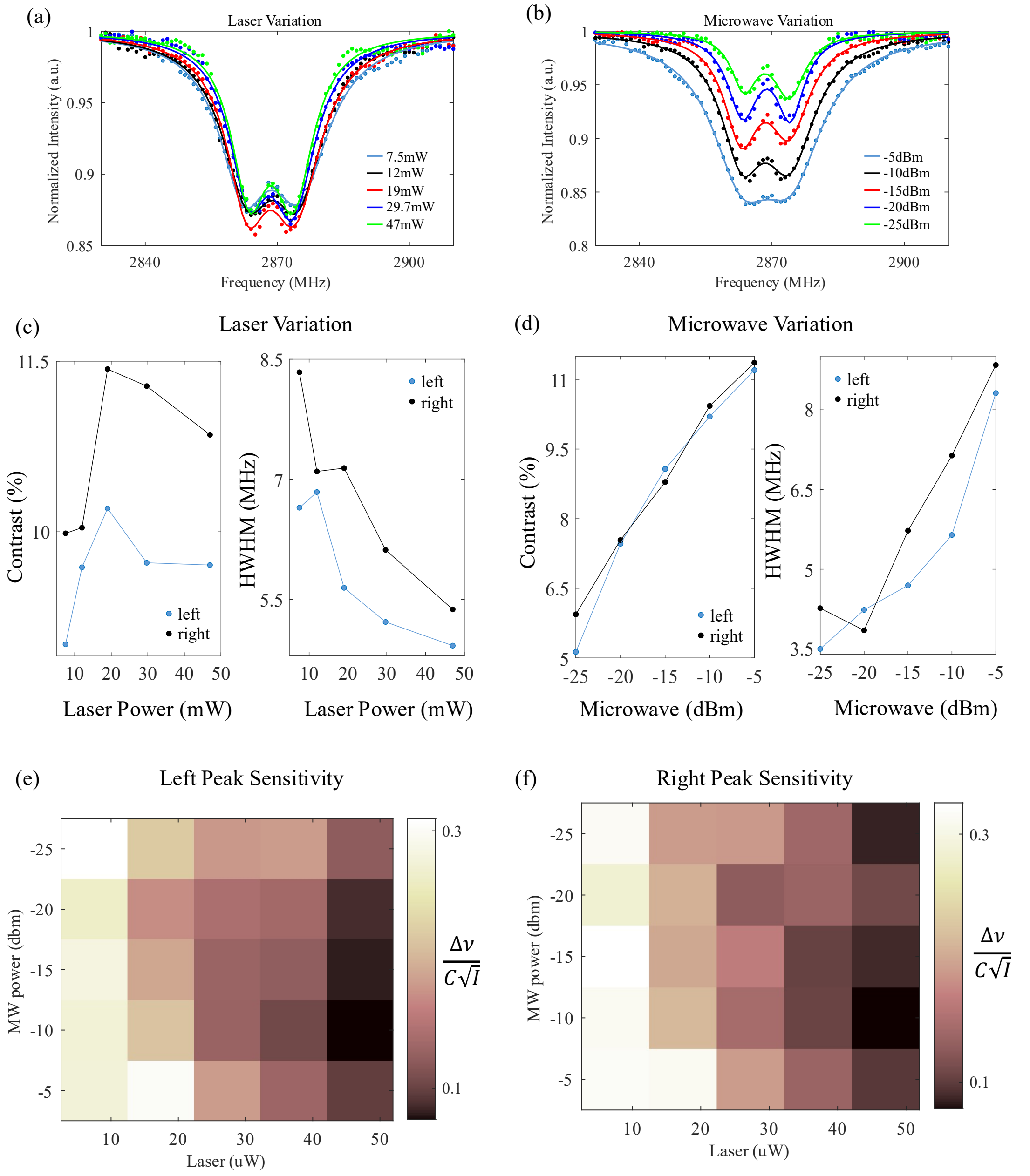

Supplementary Figure 12. Sensitivity dependence on laser and microwave powers for FNDs. **(a)** Laser power dependent ODMR, measured at a microwave power of -10 dBm from the microwave source. **(b)** Microwave power dependent ODMR, performed at a laser power of 19 mW. **(c)** Relationship between ODMR contrast/ half-width at half maximum (HWHM) Δν and laser power in (a). The ODMR contrast initially increases and then decreases as the optical power grows, while the linewidth decreases with respect to the increasing optical power. **(d)** Relationship between ODMR contrast/ HWHM Δν and microwave power in (b). Higher microwave power increases the ODMR contrast and broadens the linewidth. **(e)** Sensitivity map as a function of varying laser and microwave powers for the left peak. **(f)** Sensitivity map as a function of varying laser and microwave powers for the right peak.

The measured sensitivity map reveals a general trend: A higher microwave power requires a higher laser power to achieve optimal sensitivity. However, the maps also demonstrate that simply increasing the laser power does not necessarily yield the best sensitivity when the microwave power is fixed. Therefore, precise measurement of laser power is necessary to determine the optimal value for achieving the best sensitivity.

For our FND-receiver, the contrast in ODMR, also referred to as modulation depth, is crucial for the receiver's performance in communication systems. The FND-receiver with a higher modulation depth can serve more users and allows for more constellation points. It can also achieve a higher SNR during signal processing for recovering the information bits transmitted by users. As previously mentioned, a higher microwave power results in larger modulation depth. However, the microwave power is usually constrained by the transmitters such as mobile devices. As a result, controlling the laser power becomes the practical method at the receiver for enhancing modulation depth. When adjusting the laser power, the modulation depth initially increases but begins to decrease beyond a certain point. Notably, increased laser power also enhances NV fluorescence, leading to a higher SNR during the recovery process. Therefore, it is essential to precisely measure laser power to determine the optimal value that balances modulation depth and SNR.

Supplementary Note 5. Number of Channels in Reference-free Design

We would like to clarify the issue of number of channels from the three perspectives:

1) Key factors affecting the number of channels: The number of channels (or users) in this system is determined by the three factors. *i) Magnetic field gradients:* The applied magnetic field gradient is stated to be approximately 0.023 G/μm. This gradient causes a shift in the ODMR frequency of about 1 MHz/μm. *ii) Field of view (FOV):* The system's FOV is 75 μm × 75 μm, which defines a region where FNDs can be observed simultaneously. *iii) Frequency separation of Lorentzian peaks:* To avoid overlap in the ODMR spectrum, the Lorentzian peaks of different FNDs must be distinct. The separation between peaks depends on the axial orientation of the FNDs relative to the magnetic field gradient.

2) How 5 users were estimated: The estimation of 5 users arises from the ability to resolve distinct ODMR peaks within the given FOV and magnetic field gradient: In the experiment, we can observe 45 FNDs within the 75 μm × 75 μm FOV. The magnetic field gradient of 0.023 G/μm results in an approximate ODMR shift of 1 MHz/μm. For an FND to be resolvable in frequency space, its Lorentzian peak must not overlap with other peaks. This requires a minimum frequency separation of at least 1 MHz between neighboring FNDs. The distinct axial orientations of FNDs contribute to differences in their resonance frequencies. However, due to the randomness in axial orientations, not all FNDs in the FOV will produce sufficiently separated peaks. Out of the 45 visible spots in the FOV, only 5 FNDs are sufficiently distinct in frequency (due to spatial separation and axial orientation differences) to avoid peak overlap. This results in a utilization ratio of 5/45, meaning only 5 out of 45 available positions are usable for communication in this setup.

3) Why the utilization rate is limited to 5/45: The utilization rate is limited due to the physical and technical constraints of the system: *i) Field of view constraints:* The FOV restricts the number of observable FNDs to 45. Within this small area, the magnetic field gradient causes shifts in the ODMR peaks, but not all FNDs will have sufficiently distinct frequencies. *ii) Frequency resolution requirements:* The requirement to avoid overlapping Lorentzian peaks reduces the number of distinguishable FNDs. Even though there are 45 potential spots, only 5 FNDs meet the criteria for distinct peak separation. *iii) Axial orientation limitations:* The distinct resonance frequencies of FNDs arise from their unique axial orientations. However, the variation in these orientations is not unlimited, restricting the number of usable FNDs.

Supplementary Note 6. Scalability Issues of the System

The scalability of the system to accommodate more users would come with several trade-offs in terms of power, space, and complexity:

1) Power trade-offs: *i) Increased magnetic field strength:* To scale the system, a stronger magnetic field gradient may be required to create larger frequency separations between FNDs. This will increase power consumption. *ii) Higher optical power:* Serving more users would require detecting signals from more FNDs, necessitating higher optical power for excitation and detection, increasing energy demands.

2) Space trade-offs: *i) Larger field of view:* Accommodating more FNDs requires a larger field of view, which may increase the size of the receiver's optical and hardware components, consuming more physical space. *ii) FND density:* To fit more FNDs within the same field of view, their spatial separation must decrease, potentially reducing the distinctiveness of their resonance peaks if the applied external magnetic gradient remains unchanged.

3) Complexity trade-offs: *i) Signal demultiplexing:* As more users are added, the system must resolve more closely spaced ODMR peaks, increasing the complexity of signal processing. *ii) Alignment challenges:* With more FNDs, precise alignment and calibration of the magnetic field and optical system become more difficult.

Supplementary Note 7. Limitations of NV Receiver System

One limitation is the contrast in ODMR, which inherently limited by the physical properties of the nanodiamond itself. According to theoretical analysis and experimental results, a single NV center can exhibit a modulation depth (or contrast) of up to 30% [3]. By comparison, a large ensemble of NV centers with random orientations typically shows a much smaller contrast of approximately 1% to 2% [4]. This reduction is primarily due to the averaging effect caused by the random alignment of NV centers. We note that the FNDs used in our work generally exhibit a contrast of around 10% near their zero-field splitting frequency (Supplementary Fig. 11(b)), which has shown sufficient sensitivity for information signals detection and demultiplexing. Furthermore, the modulation depth can be improved through preferential NV alignment via material growth engineering [5]. When NV centers are predominantly aligned along a single axis, the contrast can approach 30%, thereby improving sensitivity.

Another limitation is the NV spin polarization time, which requires sufficient microwave application time to observe contrast in fluorescence. This is constrained by the transition rate between energy levels. Specifically, it refers to the k_{s0} parameter from energy level ^1E to $m_s = 0$ in the ground state ^3A shown in Supplementary Fig. 1a, with a limited rate of 0.98 ± 0.31 MHz [6]. This rate determines the bit rate, implying that the acquisition rate will be limited to approximately a maximum of 1 mega-symbols per second in a solid-state quantum system. Achieving such speeds would require faster detectors, such as high-speed cameras or single-shot detectors like photodiodes, avalanche photodiodes, or photomultiplier tubes. In addition, achieving high data rates depends on the quality of the nanodiamonds. High-quality nanodiamonds with a large number of NV centers are critical to generating sufficiently bright fluorescence signals. With adequate brightness, the effective SNR would increase, achieving the shortest acquisition time (around 1 μs) and the highest symbol rate.

Currently, our system operates with a total acquisition time of 40 ms, which includes 30 ms for camera acquisition and an additional 10 ms to trigger the microwave source. The primary factors limiting this acquisition time are listed as follows:

1) Microwave source limitations: The microwave source used in our setup (Windfreak Technology Synth HD) is unable to trigger rapidly enough to match the desired speed for changing microwave frequency or amplitude. Specifically, it requires over 5 ms to adjust the microwave parameters, even under a trigger pulse. This delay contributes to the overall acquisition time. However, this aspect pertains to the transmitter and is not a factor in our receiver's design, even though it constrains the demonstration performance.

2) Camera speed limitations: The camera used in our experiments (Teledyne Photometrics Evolve 512 Delta) is limited to a maximum frame rate of 67 frames per second, corresponding to approximately 15 ms acquisition time per frame for a full-frame pixel readout. While we could

theoretically reduce the acquisition time to around 20 ms (15 ms for the camera and 5 ms for the microwave source), this improvement would be insufficient using our current facility.

Several potential improvements could address these constraints and reduce the acquisition time:

1) Faster microwave sources: Replacing the current microwave sources with transmitters designed for commercial wireless communications could significantly reduce the delay associated with changing microwave parameters. These transmitters can achieve much faster triggering times, aligning better with high-speed requirements.

2) High-speed cameras: Utilizing event cameras, which are capable of high-speed ODMR imaging, could dramatically improve data acquisition rates. For example, event cameras have been shown to achieve image acquisition rates of up to 10 kHz [12]. This technology would enable much faster frame rates compared to our current camera.

3) Integration with photodetectors: Another approach involves integrating nanodiamonds on-chip [11] and collecting fluorescence signals using photodetectors such as photodiodes, avalanche photodiodes, or photomultiplier tubes. These detectors can achieve acquisition rates as high as 100 kHz [13,14,15]. This approach would bypass the need for slower camera-based systems, enabling real-time signal acquisition at much higher speeds.

Supplementary Note 8. Practical Analysis for 6G Requirements

We provide the practical analysis for 6G requirements from the perspectives of frequency band, bandwidth, symbol rate, channel number, crosstalk, minimum detected power, etc.

1) Frequency band: For the reference-free scheme, the operating frequency band (or bandwidth) is determined by the applied magnetic field and can be calculated using the formula: $\Gamma = \gamma B$, where γ = 28 GHz/T is the NV center gyromagnetic ratio and B refers to the magnetic field component parallel to the NV axis. Typical magnetic fields in practical implementations can easily reach several Tesla, resulting in bandwidths ranging from several GHz to hundreds of GHz. This capability positions the NV center-based system to support millimeter-wave bands (30–300 GHz), which are envisioned as the working band for 6G networks [7]. For the reference-based scheme, the NV center operates at its intrinsic spin resonance frequency of 2.87 GHz. To extend the system to higher frequency bands, such as millimeter-wave or terahertz bands, a frequency mixer can be utilized to down-convert the target microwave signals into the NV center's detection range. Recent studies have demonstrated the feasibility of such an approach, achieving broadband microwave detection using spins in diamond interfaced with a thin-film magnet [8]. This method allows the system to support higher frequency bands, potentially extending into the terahertz range (0.1–10 THz) depending on the mixer's performance and design.

2) Bandwidth: For the reference-based approach, the current bandwidth is determined by the linewidth of the ODMR, which is approximately 6–7 MHz. This represents an inherent limitation of the NV center's physical properties. For the reference-free approach, the bandwidth is determined by the applied external static magnetic field, which can be calculated using the formula: $\Gamma = \gamma B$, where γ = 28 GHz/T is the NV center gyromagnetic ratio and B refers to the magnetic field component parallel to the NV axis. In practical systems, magnetic fields can easily reach several Tesla, resulting in bandwidths ranging from tens of GHz to hundreds of GHz. This large bandwidth is sufficient to meet the requirements of next-generation 6G networks in mid-band spectrum [9]. However, if future demands were to require bandwidths in the THz range, such requirements would exceed the capabilities of the NV center-based receiver system. In such cases, alternative quantum systems with broader bandwidths could be explored. While our current study focuses on NV centers, we believe our approach demonstrates the feasibility of integrating general quantum systems into wireless communication systems, paving the way for further advancements in this area.

3) Symbol rate: The current data rate in our experiments is constrained by hardware limitations, specifically the speed of the microwave source and the camera, resulting in a minimum acquisition time of approximately 20 ms. Once these hardware constraints are addressed, the data rate will instead be limited by the interaction time between the microwave field and the spin states of NV centers in the receiver. For continuous-wave (CW) ODMR, the NV spin polarization time requires sufficient microwave application time to observe contrast in fluorescence. This is constrained by the

transition rate between energy levels. Specifically, it refers to the k_{s0} parameter, with a limited rate of 0.98 ± 0.31 MHz [6]. This rate determines the bit rate, implying that the acquisition rate will be limited to approximately a maximum of 1 mega-symbols per second in a solid-state quantum system. Achieving such speeds would require faster detectors, such as high-speed cameras or single-shot detectors like photodiodes, avalanche photodiodes, or photomultiplier tubes. In addition, achieving high data rates depends on the quality of the nanodiamonds. High-quality nanodiamonds with a large number of NV centers are critical to generating sufficiently bright fluorescence signals. With adequate brightness, the effective SNR would increase, achieving the shortest acquisition time (around 1 μs) and the highest symbol rate. Since 6G is still under development and lacks standardization, we can compare our system to the current 5G standard. According to the 5G New Radio (NR) standards outlined in 3GPP [10], a radio frame is fixed at 10 ms and consists of 10 subframes, each lasting 1 ms. Each subframe contains 1–16 slots, with each slot comprising 14 symbols. This results in a symbol duration ranging from 4.46 to 71.43 μs, corresponding to a symbol rate of 0.014 to 0.22 mega-symbols per second. Notably, the potential symbol rate of FND-receiver (1 mega-symbols per second) exceeds that of current traditional receivers (0.014-0.22 mega-symbols per second) based on the 5G NR standards [10].

4) Channel number: Channel number is a critical parameter for multi-user communication in 6G networks. In our current setup, for the reference-free scheme, the channel number is determined by the number of distinct microwave frequencies that can be addressed simultaneously without overlap. Here, the magnetic field gradient creates a frequency separation between NV centers across the field of view, allowing multiple channels to coexist. The number of channels is limited by the available bandwidth provided by the magnetic field (up to hundreds of GHz) and the linewidth of the ODMR signal (approximately 6–7 MHz). In our experiment, 5 out of 45 FNDs can be distinguished, resulting in a utilization ratio of 11.1%. Thus, when we broaden the field of view to thousands of FNDs, we can accommodate up to hundreds of users which is sufficient for ubiquitous radio access in 6G network. For the reference-based scheme, the channel number could be increased by employing multiplexing techniques, such as spatial-division multiplexing by spatially separating NV centers using patterned fields or multiple spatially resolved detectors. Future work could focus on enhancing channel number by optimizing the magnetic field gradient and the NV center alignment.

5) Crosstalk: Crosstalk occurs when signals from adjacent channels interfere with one another, degrading the system performance. In our NV center-based system, crosstalk is primarily influenced by the linewidth of the ODMR signal and the precision of frequency separation. For the reference-free scheme, the use of a magnetic field gradient reduces crosstalk by separating NV center resonance frequencies spatially and spectrally. In the reference-based scheme, employing a high-quality narrowband filtering can minimize interference between channels. Additionally, the quantum nature of NV centers enables highly precise signal processing, which inherently reduces

crosstalk compared to classical systems.

6) Minimum detected power: The dynamic range of the microwave power in our system spans approximately 30 dBm. The minimum microwave power detectable by the system is -25 dBm at the microwave source. The 6G systems are expected to operate with extremely low power levels, particularly for IoT devices and energy-efficient applications. While our current system demonstrates a reasonable dynamic range, further improvements to sensitivity may be required to meet the stringent power efficiency standards of 6G. Enhancing the SNR through high-quality NV centers, optimized microwave amplifiers, and advanced detection techniques (e.g., lock-in detection) could lower the minimum detected power threshold.

7) Suitability for 6G networks: We would clarify that different 6G application scenarios have distinct requirements in terms of latency, data rate, bandwidth, etc. Our system is specifically designed to target the scenario of ubiquitous radio access in the 6G networks. In comparison to state-of-the-art communication systems, our NV center-based receiver system shows significant promise for integration into 6G networks. Traditional receiver designs are limited in size due to the requisite half-wavelength spacing between antenna elements, and restricted bandwidth which necessitates multiple RF receivers for multi-band signals detection. Our design addresses these issues by offering the key advantages: NV centers operate in the quantum domain and exhibit greater sensitivity than traditional metal antennas, almost have **no spacing constraints**. This makes them promising candidates for developing compact and sensitive receivers. Moreover, in contrast to traditional receivers with components like RF antennas, analog filters, and mixers, NV-based receivers or sensors mainly use optical components for detection and down-conversion, marking them **inherently immune to electrical noise**. In summary, the key advantages include: *i) Wide bandwidth:* The system can naturally support millimeter-wave bands and can be extended into terahertz frequencies using a frequency mixer. *ii) Quantum-enhanced properties:* NV centers offer unique advantages, such as high sensitivity and immunity to electrical noise, which could provide an edge over classical systems in specific applications.

Supplementary Note 9. Size, Weight, and Power Consumption

Here are the details of the size, weight, and power consumption (SWaP-C) of our implemented receiver.

1) SWaP-C breakdown and improvements:

i) Size: Our current receiver prototype measures 300 mm × 300 mm × 200 mm, encompassing all optical and electronic components. The size can be significantly reduced by integrating the optical and electronic components into a chip-scale platform [11], minimizing reliance on bulky breadboards and external devices. On-chip integration of FNDs and fiber-coupled excitation of NV centers would make the system more portable and scalable.

ii) Weight: The total weight is approximately 8 kg, with optical breadboards contributing 6 kg and the remaining optical and electronic components weighing below 2 kg. Replacing heavy breadboards with compact, purpose-built optical mounts or chip-scale photonic devices would drastically reduce weight. With full on-chip integration and the removal of breadboards, the weight can be greatly reduced, enabling deployment in portable or even handheld devices.

iii) Power consumption: In our receiver prototype, the power consumption for the camera and laser are 1.17 W and 0.7 W respectively, resulting in a total power consumption of 1.87 W. Replacing the camera with more efficient optical detectors can reduce power consumption significantly. Advanced laser designs with lower power thresholds can further reduce optical component power requirements. Integrating FNDs on-chip can operate efficiently with laser power levels around 100 μW [11].

2) Potential: *i) Miniaturization:* On-chip integration of FNDs and fibers would allow for highly compact, lightweight, and low-power modules suitable for large-scale deployment in portable devices and networks. *ii) Cost reduction:* Once integrated into scalable semiconductor or photonic platforms, the cost of manufacturing and deploying these devices would decrease significantly. *iii) Versatility:* The system's ability to operate at low power and detect weak signals makes it ideal for applications in ubiquitous radio access wireless systems.

3) Limitations: *i) Fabrication challenges:* Incorporating FNDs into photonic integrated circuits while maintaining high operational efficiency and sensitivity requires precise fabrication techniques. *ii) Material availability and processing:* Integration of FNDs into scalable platforms (e.g., silicon photonics) might require additional material-processing steps, increasing complexity. *iii) Advanced laser design:* The reduction of power consumption requires advanced laser designs with lower power thresholds, which is challenging to maintain the quality of laser at the same time.

Supplementary References:

[1] A. Dréau, M. Lesik, L. Rondin, P. Spinicelli, O. Arcizet, J-F. Roch, and V. Jacques. “Avoiding power broadening in optically detected magnetic resonance of single NV defects for enhanced dc magnetic field sensitivity.” *Physical Review B—Condensed Matter and Materials Physics* 84, no. 19 (2011): 195204.

[2] P. Wang, Z. Yuan, P. Huang, X. Rong, M. Wang, X. Xu, C. Duan, C. Ju, F. Shi, and J. Du. “High-resolution vector microwave magnetometry based on solid-state spins in diamond.” *Nature Communications* 6, no. 1 (2015): 6631.

[3] F. Jelezko, and J. Wrachtrup. “Single defect centres in diamond: A review.” *Physica Status Solidi (a)* 203, no. 13 (2006): 3207-3225.

[4] A. FL Poulsen, J. D. Clement, J. L. Webb, R. H. Jensen, L. Troise, K. Berg-Sørensen, A. Huck, and U. L. Andersen. “Optimal control of a nitrogen-vacancy spin ensemble in diamond for sensing in the pulsed domain.” *Physical Review B* 106, no. 1 (2022): 014202.

[5] C. Osterkamp, M. Mangold, J. Lang, P. Balasubramanian, T. Teraji, B. Naydenov, and F. Jelezko. “Engineering preferentially-aligned nitrogen-vacancy centre ensembles in CVD grown diamond.” *Scientific Reports* 9, no. 1 (2019): 5786.

[6] J. Klatzow, J. N. Becker, P. M. Ledingham, C. Weinzetl, K. T. Kaczmarek, D. J. Saunders, J. Nunn, I. A. Walmsley, R. Uzdin, and E. Poem. “Experimental demonstration of quantum effects in the operation of microscopic heat engines.” *Physical Review Letters* 122, no. 11 (2019): 110601.

[7] W. Hong, Z.-H. Jiang, C. Yu, D. Hou, H. Wang, C. Guo, Y. Hu, L. Kuai, Y. Yu, Z. Jiang, Z. Chen. "The role of millimeter-wave technologies in 5G/6G wireless communications." *IEEE Journal of Microwaves* 1, no. 1 (2021): 101-122.

[8] J. J. Carmiggelt, I. Bertelli, R. W. Mulder, A. Teepe, M. Elyasi, B. G. Simon, G. EW Bauer, Y. M. Blanter, and T. van der Sar. "Broadband microwave detection using electron spins in a hybrid diamond-magnet sensor chip." *Nature Communications* 14, no. 1 (2023): 490.

[9] Qualcomm Whitepaper, “Vision, market drivers, and research directions on the path to 6G,” Dec. 2022. [Online] https://www.qualcomm.com/content/dam/qcomm-martech/dm-assets/documents/Qualcomm-Whitepaper-Vision-market-drivers-and-research-directions-on-the-path-to-6G.pdf

[10] 3GPP TS 38.211, “NR; Physical channels and modulation,” Release 16, V16.4.0, Table 4.3.2-1, 2020-12.

[11] H. Siampour, S. Kumar, V. A. Davydov, L. F. Kulikova, V. N. Agafonov, and S. I. Bozhevolnyi. "On-chip excitation of single germanium vacancies in nanodiamonds embedded in plasmonic

waveguides." *Light: Science & Applications* 7, no. 1 (2018): 61.

[12] Z. Du, M. Gupta, F. Xu, K. Zhang, J. Zhang, Y. Zhou, Y. Liu et al. "Widefield Diamond Quantum Sensing with Neuromorphic Vision Sensors." *Advanced Science* 11, no. 2 (2024): 2304355.

[13] D. Le Sage, L. My Pham, N. Bar-Gill, C. Belthangady, M. D. Lukin, A. Yacoby, and R. L. Walsworth. "Efficient photon detection from color centers in a diamond optical waveguide." *Physical Review B—Condensed Matter and Materials Physics* 85, no. 12 (2012): 121202.

[14] Q. Gu, L. Shanahan, J. W. Hart, S. Belser, N. Shofer, M. Atature, and H. S. Knowles. "Simultaneous nanorheometry and nanothermometry using intracellular diamond quantum sensors." *ACS Nano* 17, no. 20 (2023): 20034-20042.

[15] J. Yun, K. Kim, S. Park, and D. Kim. "Temperature Selective Thermometry with Sub-Microsecond Time Resolution Using Dressed-Spin States in Diamond." *Advanced Quantum Technologies* 4, no. 11 (2021): 2100084.
